# Transcriptome-Based Analysis of the Effects of Salt Stress on the Embryos of *Hypomesus nipponensis*

**DOI:** 10.3390/ani16182910

**Published:** 2026-09-16

**Authors:** Qian Xiao, Xinnan Fu, Sitong Li, Zhelan Wang, Shuhan Chen, Yang Cao, Kai Deng, Junjie Zhang

**Affiliations:** 1 College of Life Sciences, Xinjiang Agricultural University, Urumqi 830052, China; qianxiao181@163.com (Q.X.); 18087035850@163.com (X.F.); 18160510177@163.com (S.L.); m15286133601@163.com (Z.W.);; 2 Xinjiang Key Laboratory for Ecological Adaptation and Evolution of Extreme Environment Organisms, College of Life Sciences, Xinjiang Agricultural University, Urumqi 830052, China; 3 College of Modern Fishery Industry, Xinjiang Agricultural University, Urumqi 830052, China; 4 Xinjiang Characteristic Aquatic Research Center, Urumqi 830052, China; 5 Xinjiang Bosteng Lake Ecological Fisheries Co., Ltd., Bohu 841400, China

**Keywords:** *Hypomesus nipponensis*, embryo, salinity stress, transcriptome, differentially expressed genes, salinity tolerance

## Abstract

The increasing salinization and alkalization of inland waters in China pose a growing threat to the early developmental stages of fish. This study investigated the survival rates and transcriptomic response mechanisms of pond smelt (*Hypomesus nipponensis*) embryos, a species naturally adapted to brackish water, under different salinity stresses. Fertilized eggs were exposed to four salinity gradients: freshwater (0‰, experimental control) and 7‰, 14‰, and 21‰ for 7 days. The results showed that the upper salinity tolerance limit for embryos was between 21‰ and 28‰; hatching rates were zero in the 28‰ and 35‰ groups, and the hatching rate in the 21‰ group decreased to 40.3 ± 6.1%. Transcriptome sequencing analysis of the 0‰, 7‰, 14‰, and 21‰ groups revealed that salinity stress induced extensive differential gene expression in a non-monotonic response pattern. In the 7‰ and 14‰ groups, the number of downregulated genes (approximately 10,000) was significantly higher than that of upregulated genes (approximately 3300). In contrast, in the high-salinity 21‰ group, upregulated genes increased to 4535. Functional enrichment analysis revealed that the calcium signaling pathway, axon guidance, and glutamatergic synapse pathway were significantly enriched across all salinity groups. Changes in the expression of key differential genes (e.g., *sparc*, *hspa4a*, *aldob*, and *csmd1a*) further confirmed molecular events such as dysregulation of calcium homeostasis, stress protein activation, metabolic inhibition, and neurodevelopmental regulation. The qRT-PCR validation results were highly consistent with the transcriptome data. This study is the first to systematically reveal the molecular regulatory network of pond smelt embryos in response to increasing salinity relative to a freshwater reference point. Our findings indicate their potential for proliferation in moderate saline–alkaline waters (≤14‰), while high salinity (≥21‰) significantly reduces hatching success and triggers a strong stress response. These results provide an important molecular basis and theoretical reference for the enhancement and aquaculture of this species in saline–alkaline waters.

## Simple Summary

Climate change and human activities are increasing the salinity of many inland waters, which poses a threat to fish reproduction. The pond smelt (*Hypomesus nipponensis*) is a fish that naturally inhabits brackish water, making it a valuable model for studying how fish cope with salt stress. In this study, we exposed pond smelt embryos to four salinity levels, ranging from freshwater to 21 parts per thousand (‰), for seven days and examined the resulting changes in their gene activity. We found that the embryos could tolerate up to 21‰ salinity, although their hatching success decreased at higher salinities. Salt stress caused thousands of genes to alter their activity. At moderate salinities, more genes were downregulated than upregulated, but at the highest salinity, the number of upregulated genes increased. Our results provide a molecular basis for evaluating the potential of this fish for aquaculture in saline or alkaline waters, which could aid in utilizing vast, currently unused, saline water resources.

## 1. Introduction

*Hypomesus nipponensis*, commonly known as the pond smelt or cucumber fish, belongs to the genus Hypomesus within the family Osmeridae (order Salmoniformes) and is a small, annual commercial fish. It is native to Hokkaido, Japan, and was later introduced to the Yalu and Tumen Rivers in China. It has now been widely distributed in reservoirs and lakes in provinces such as Xinjiang, Heilongjiang, and Yunnan. This species inhabits low-temperature waters, adapts to both freshwater and brackish environments (predominantly brackish water), and exhibits anadromous migratory behavior. It reaches sexual maturity at one year of age, with its spawning period occurring from March to May. In China, *H. nipponensis* has formed dominant populations in waters such as Bosten Lake in Xinjiang, making it an important local fishery resource [1,2].

The chemical types of saline–alkaline water bodies in China are complex and diverse, with significant regional differences in ion composition. In arid and semi-arid climatic zones, evaporation far exceeds precipitation, causing soluble salts in the soil to continuously accumulate on the surface through capillary action. This forms sulfate–chloride-type saline–alkaline water bodies (accounting for approximately 96.1% of the national saline–alkaline land). In Northeast China, carbonate-type soda saline–alkaline landforms are dominant (around 3.2%), while eastern coastal areas are dominated by chloride-type water bodies (less than 1%). The low-lying saline–alkaline waters associated with these lands cover approximately 690 million mu (about 46 million hectares), of which over 90% remains unused. This diversity in water chemistry types implies that aquaculture suitability plans developed for one water body cannot be directly transferred to another, necessitating salinity adaptation studies for specific species. As a species naturally adapted to brackish water, *H. nipponensis* serves as an ideal model for studying the proliferation potential of fish in saline–alkaline waters [3,4].

Salinity is a key environmental factor affecting fish growth, development, reproduction, and distribution [3,5]. With global climate change and human activities, the salinization and alkalization of inland waters are intensifying, posing severe challenges to the survival and reproduction of brackish and freshwater fish. The embryonic stage is one of the most sensitive developmental stages to environmental stress in the fish life cycle. Salinity stress not only affects embryo survival but may also affect long-term growth and development [6]. For instance, studies have shown that Nile tilapia (*Oreochromis niloticus*) embryos can still hatch at salinities of up to 20 ppt [4,7]; therefore, assessing the salinity tolerance of *H. nipponensis* embryos and elucidating the underlying molecular response mechanisms hold significant theoretical and applied value.

To date, numerous transcriptomic studies have been conducted to investigate the molecular responses of diverse aquatic species, including both fish and crustaceans, to salinity and alkalinity stress. These studies have revealed that exposure to ionic stress induces extensive changes in gene expression profiles, with differentially expressed genes frequently enriched in pathways associated with immune regulation, metabolic processes, and signal transduction. Collectively, these findings suggest that adaptation to saline–alkaline environments involves the coordinated regulation of multiple physiological processes, such as ion transport, lipid metabolism, apoptosis, and immune function [8,9,10,11,12,13,14,15,16].

Despite extensive research on the molecular mechanisms of salinity stress in fish, transcriptomic studies on *H. nipponensis*—particularly at the embryonic stage—and its potential for salinity tolerance are lacking. In this study, *H. nipponensis* embryos exposed to different salinities (freshwater 0‰ as the experimental reference and 7‰, 14‰, 21‰, 28‰, and 35‰ salinity) were used for physiological observation; the 0‰, 7‰, 14‰, and 21‰ groups underwent transcriptome sequencing (the 28‰ and 35‰ groups were used solely to determine the upper salinity tolerance limit and were not sequenced) to systematically analyze the effects of salinity stress on embryonic gene expression. GO functional annotation and KEGG pathway enrichment analyses were performed to elucidate the molecular response mechanisms. Based on molecular evidence, the reproductive potential of this species was evaluated comprehensively in high-salinity waters. We aimed to provide scientific evidence for the development of fishery resources in saline–alkaline waters.

## 2. Materials and Methods

### 2.1. Source of Experimental Fish and Artificial Fertilization

Parental fish were captured on 16 March 2025 from the Kaidu River in Bohu County, Bayingolin Mongol Autonomous Prefecture, Xinjiang Uygur Autonomous Region, China. Healthy individuals with intact morphology were selected for artificial fertilization. Eggs and sperm were collected from 50 females and 50 males, respectively, by gently squeezing the abdomen and collecting the gametes into a dry basin. The gametes were then gently mixed using a dry feather. Subsequently, an appropriate amount of purified water was added, and the mixture was gently shaken to ensure thorough mixing of sperm and eggs. Successful fertilization was confirmed by observing embryo development to the eyed stage, and successful hatching was defined by the complete emergence of larvae from the chorion. The resulting fertilized eggs were used for the subsequent stress experiments.

### 2.2. Experimental Design and Stress Treatment

Immediately after artificial fertilization, the fertilized eggs were transferred to various salinity solutions to initiate stress exposure. Four salinity treatment levels were prepared by dissolving artificial sea salt in river water: freshwater control (0‰, designated S1), 7‰ (S2), 14‰ (S3), and 21‰ (S4). Two additional salinity treatments, 28‰ (S5) and 35‰ (S6), were also established; these groups were used solely to observe hatching rates to determine the upper salinity tolerance limit and were not subjected to transcriptome sequencing analysis. Salinity was measured using a salinometer, and pH was measured using a pH meter to ensure consistent alkalinity. The control group used locally produced purified water (pH 7.2), while the salinity treatment groups used natural river water (pH 7.5) for preparation. The similar pH values were confirmed before the experiment. However, the purified water contains an extremely low mineral ion content, which fundamentally differs in ionic composition from natural river water. This difference in background water chemistry is a significant confounding factor to consider when interpreting the results of this experiment. Each salinity treatment had three biological replicates, and stress exposure lasted for 7 days.

Fertilized eggs were evenly spread on custom-made polyethylene plastic frames (10 cm × 10 cm × 5 cm, length × width × height), with the bottom and sides lined with 60-mesh (aperture ≈ 0.25 mm) nylon netting, forming incubation baskets. Each basket was placed in an individual cylindrical polyethylene bucket (diameter 50 cm and height 55 cm) containing the corresponding salinity solution, with the water level adjusted to completely submerge the basket. This ensured full contact of the embryos with the water. Each bucket was one experimental unit, and all buckets were kept in the same constant-temperature environment. During the experiment, one-third of the water was replaced daily, and dissolved oxygen was maintained above 6 mg/L to ensure basic conditions for normal embryonic development.

For each replicate bucket, the total number of initially stocked fertilized eggs (N_total_) was recorded (approximately 300–500 eggs per bucket). On day 6 of stress, the number of embryos that had developed to the eyed stage (N_eyed_) was counted under a dissecting microscope. On day 8, the number of larvae that had completely hatched (N_hatched_) was recorded. The fertilization rate and hatching rate for each replicate were then calculated as follows:

$$Fertilization\;rate\;(\%)=\frac{N_{eyed}}{N_{total}}\times 100\%$$

$$Hatching\;rate\;(\%)=\frac{N_{hatched}}{N_{total}}\times 100\%$$

Final fertilization and hatching rates for each salinity treatment are expressed as the mean ± standard deviation (SD) of the three biological replicates.

On day 7 of stress treatment, the fertilization rate (using embryo eyeing as an indicator of completed fertilization) was recorded. Embryo samples were collected, immediately frozen in liquid nitrogen, and stored for subsequent RNA extraction and transcriptome sequencing. Hatching rates were recorded on day 8 of stress.

Each salinity group comprised three replicate buckets (i.e., three biological replicates, *n* = 3). Each bucket contained one incubation basket with approximately 300–500 fertilized eggs for fertilization and hatching rate statistics (physiological indicators). On day 7 of stress treatment, for transcriptome sequencing, 30 surviving embryos at a consistent developmental stage were randomly selected from each bucket (i.e., each biological replicate) and pooled as one RNA-seq sample. Thus, each salinity group had three RNA-seq biological replicates (*n* = 3), totaling 12 sequencing samples.

All physiological data (fertilization and hatching rates) and qRT-PCR data are expressed as mean ± SD. Statistical comparisons among multiple salinity groups were performed using one-way analysis of variance (ANOVA) followed by Tukey’s post hoc test. Comparisons between two groups were performed using Student’s *t*-test. A probability level of *p* < 0.05 was considered statistically significant. All statistical analyses were conducted using GraphPad Prism 10.0.

### 2.3. Transcriptome Sequencing and Data Analysis

Total RNA was extracted from each of the three biological replicates per group using the TRNzol Universal Total RNA Extraction Kit (Tiangen Biotech (Beijing, China) Co., Ltd., Cat# DP424). cDNA libraries were constructed after RNA quality assessment. Transcriptome sequencing was completed by Shanghai Lingen Biotechnology Co., Ltd. (Shanghai, China). using the DNBSEQ-T7 high-throughput sequencing platform (paired-end 150 bp reads).

Raw sequencing data were quality-controlled using Trimmomatic (v0.39) to remove adapter sequences, trim low-quality bases (quality value < 20), and filter reads with N ratio > 10% or length < 75 bp. Clean reads were aligned to the *H. nipponensis* reference genome (genome size: 538,634,120 bp; 80 scaffolds; GC content: 45.86%) using Hisat2 (v2.1.0). Overall RNA-seq quality assessment was performed using QualiMap (v2.2.1). Gene expression levels were quantified using featureCounts (v2.0.1), and normalized expression values were calculated as FPKM (Fragments Per Kilobase of Transcript Per Million Mapped Reads) and TPM (Transcripts Per Million) [17,18].

Differential expression analysis was performed using the R package edgeR (v3.36.0). For the initial screening of genome-wide differentially expressed genes (DEGs), thresholds were set as |log_2_(fold change)| ≥ 1 and FDR < 0.05. For subsequent downstream analyses (GO and KEGG enrichment and key gene screening), a more stringent threshold of |log_2_FC| ≥ 2 and FDR < 0.05 was applied to ensure high biological robustness of the reported pathways and genes. GO functional enrichment was performed using Goatools (0.9.9), and KEGG pathway enrichment was performed using KOBAS (v3.0). Enriched terms and pathways were considered significant at *p* < 0.05 [19].

### 2.4. Real-Time Quantitative PCR Validation

To validate the reliability of the transcriptome data, six differentially expressed genes (*GRPEL1*, *CSMD1A*, *SPARC*, *HNRNPA0*, *ALDOB*, *HSPA4A*) were randomly selected for real-time quantitative PCR (qRT-PCR) analysis. Total RNA was extracted from embryo samples for each salinity group using the TRNzol Universal Total RNA Extraction Kit (Tiangen Biotech (Beijing, China) Co., Ltd., Cat# DP424), and cDNA was synthesized by reverse transcription using the FastKing RT Kit (with gDNA removal, Tiangen, Beijing, China, Cat# KR116) according to the manufacturer’s instructions. qRT-PCR was performed using Takara’s SYBR^®^ Premix Ex Taq™ II (Tli RNaseH Plus, Kusatsu, Japan, RR820A) on a CFX96 real-time PCR detection system (Bio-Rad, Hercules, CA, USA). The reaction mixture (total volume 20 μL) consisted of SYBR^®^ Premix Ex Taq™ II (2×) 10 μL, forward and reverse primers (10 μM) 0.8 μL each, cDNA template 2 μL, and nuclease-free water 6.4 μL. The thermal cycling program was 95 °C for 30 s for initial denaturation, followed by 40 cycles of 95 °C for 5 s and 60 °C for 34 s for annealing and extension. Melt curve analysis was performed after each run to confirm amplification specificity. β-actin was used as the internal reference gene, which has been widely validated as a stably expressed housekeeping gene across different developmental stages and tissue types in fish and confirmed under our experimental conditions (Ct value coefficient of variation < 5% across treatment groups in pre-tests). All reactions were performed in triplicate technical replicates, and relative expression levels were calculated using the 2^−ΔΔCt^ method [20]. Primers were synthesized by Sangon Biotech (Shanghai, China) Co., Ltd., and their sequences are listed in Table 1. qRT-PCR results were compared with transcriptome data to validate the reliability of the RNA-seq analysis.

## 3. Results

### 3.1. Effects of Salinity Stress on Fertilization and Hatching Rates

**Figure 1:**
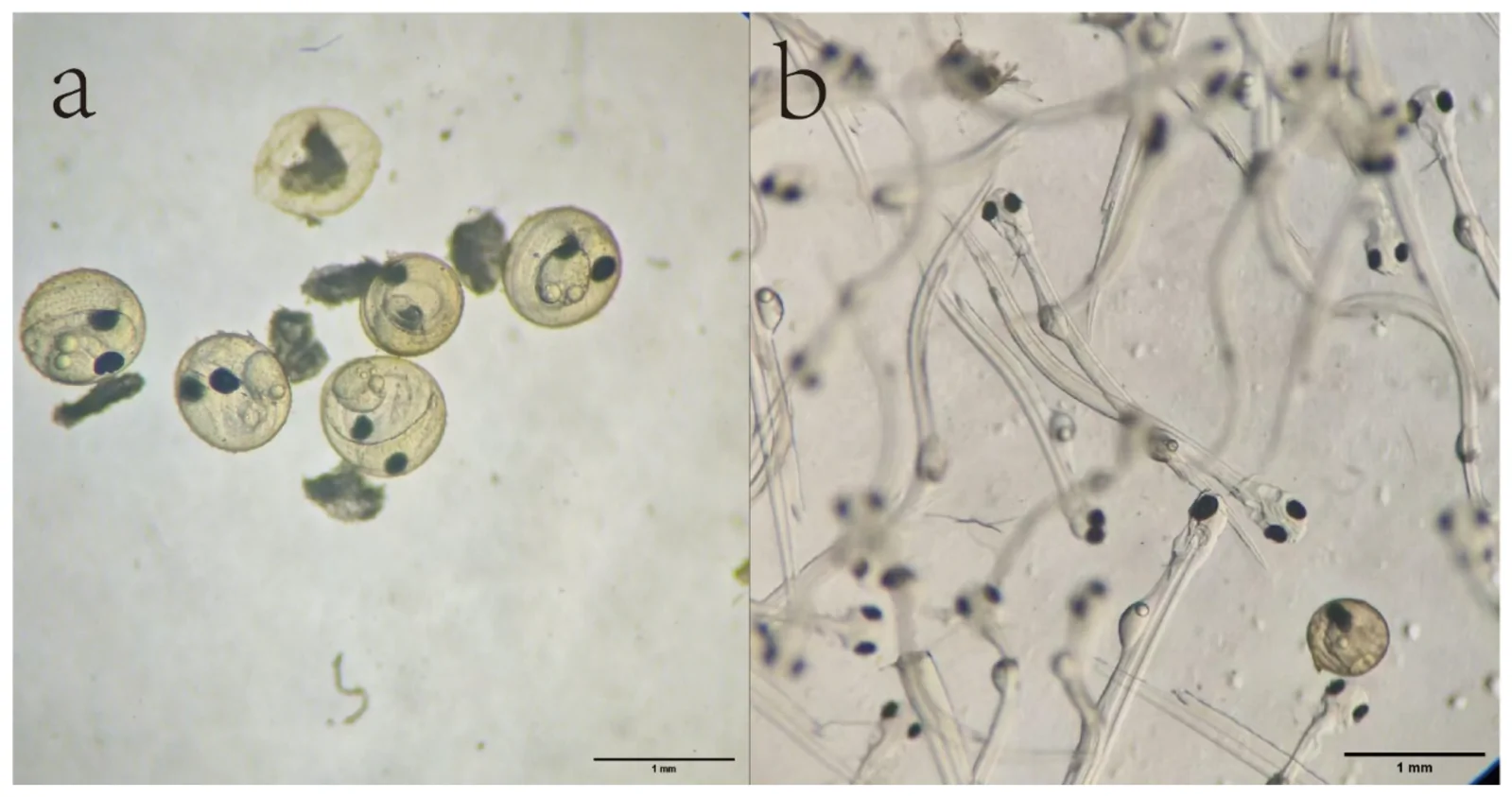
(**a**) Fertilized egg of *H. nipponensis* (eye pigment stage); (**b**) newly hatched larva.

**Figure 2:**
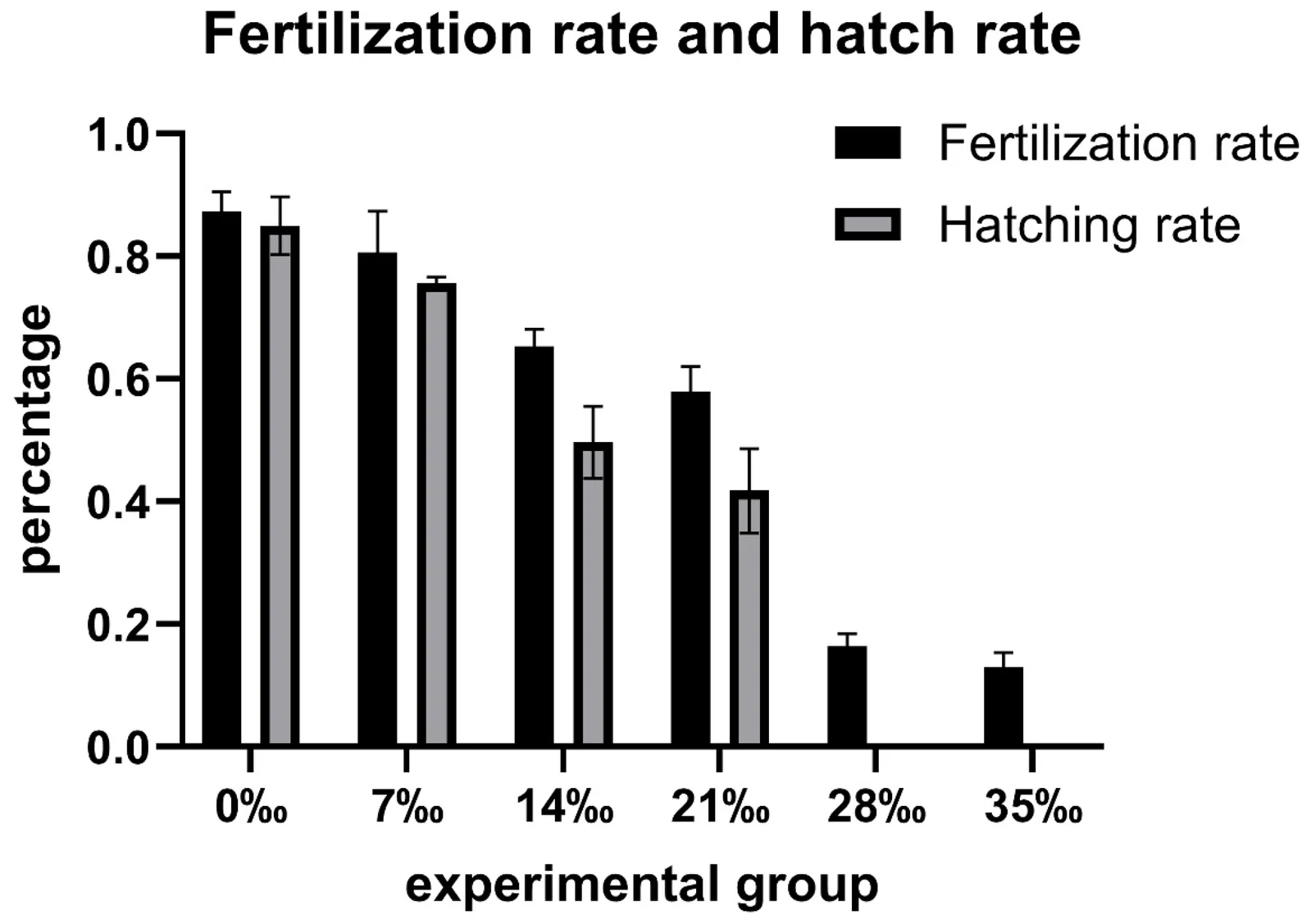
Fertilization and hatching rates of *H. nipponensis* under salinity stress.

To evaluate the survival and developmental capacity of *H. nipponensis* embryos, fertilization and hatching rates were recorded for each salinity group (Figure 1 and Figure 2). In the freshwater control group (0‰), the average fertilization rate was (87.1 ± 1.9)%, and the hatching rate was (80.1 ± 1.7)%, indicating good egg quality. As salinity increased to 7‰ and 14‰, fertilization rates slightly decreased but remained relatively high (85.0% and 75.5%, respectively), while hatching rates decreased to (74.5 ± 4.1)% and (48.3 ± 4.5)%, respectively. At 21‰ salinity, the fertilization rate dropped to (56.8 ± 3.7)%, and the hatching rate further decreased to (40.3 ± 6.1)%. Notably, at 28‰ and 35‰ salinity, although some fertilized eggs were still observed (16.7% and 11.9%, respectively), hatching rates were zero, indicating that embryos failed to develop normally. Because only these two salinity groups were used to determine the upper tolerance limit and were not sequenced, subsequent transcriptome analysis focused only on the S1 (0‰), S2 (7‰), S3 (14‰), and S4 (21‰) groups. These results indicate that the upper salinity tolerance limit for *H. nipponensis* embryos is between 21‰ and 28‰, and the hatching rate is more sensitive to salinity than the fertilization rate (Figure 2).

### 3.2. Quality Control of Raw Sequencing Data

Second-generation sequencing was performed, and raw data were processed using Trimmomatic software. A single run of second-generation sequencing can generate billions of reads; it is impractical to display the quality of each read individually. By using statistical methods to analyze base distribution and quality fluctuations for each cycle of all sequenced reads, the sequencing quality and library construction quality of the samples can be intuitively reflected macroscopically. Raw sequencing data contain adapter sequences, low-quality reads, sequences with high N ratios, and reads that are too short, which seriously affect the quality of subsequent assembly.

**Figure 3:**
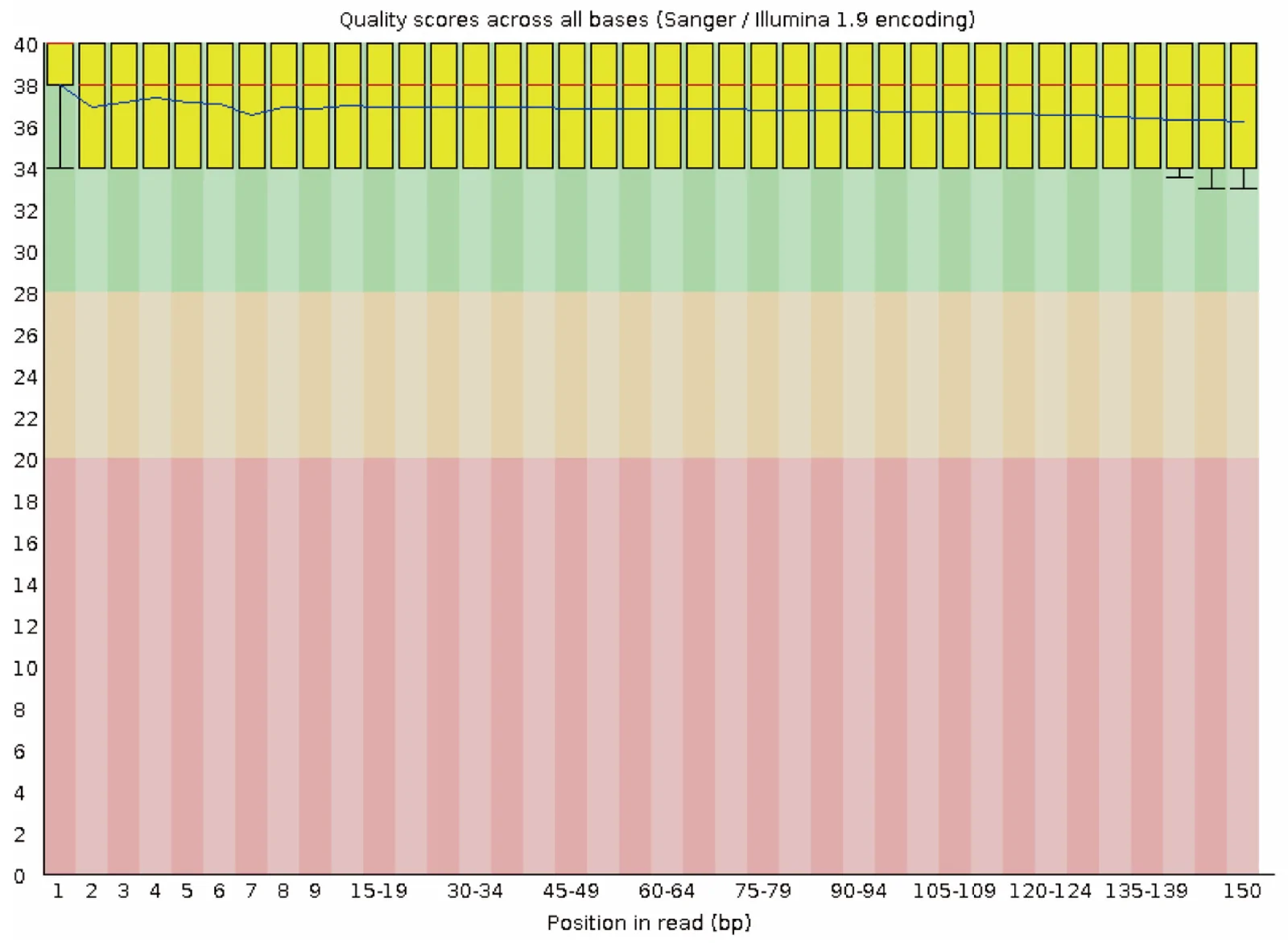
Read base quality distribution plot. Note: The x-axis represents the read base coordinates, indicating the sequential arrangement of bases from the 5′ to 3′ end of the reads. The y-axis represents the base quality score of the reads (Q score; Solexa scale: 40 = highest, −15 = lowest). If a base has a quality score of 30 (Q30), the prob-ability of a sequencing error at that base is 0.001 (10^−3^). The box plot represents the distribution of quality scores of all bases at each position. The middle red line represents the median; the upper and lower edges of the yellow-filled box represent the upper and lower quartiles, respectively. The upper whisker represents the 90th percentile, and the lower whisker represents the 10th percentile. The blue line represents the mean. The background colors from top to bottom are green, orange, and red, representing very good, reasonable, and poor, respectively, dividing base quality into three different categories.

To ensure the accuracy of subsequent bioinformatics analyses, raw sequencing data were first filtered to obtain high-quality clean data. The specific steps and order were as follows: (1) Remove adapter sequences from reads, and remove reads with no insert fragments due to adapter self-ligation, etc. (2) Trim low-quality (quality value < 20) bases from the 3′ end of sequences. If the remaining sequence still has bases with a quality value < 10, discard the entire read; otherwise, keep it. (3) Remove reads with a N ratio exceeding 10%. (4) Discard sequences with a length less than 75 bp after adapter removal and quality trimming (Figure 3).

### 3.3. Statistics of Differentially Expressed Genes

**Figure 4:**
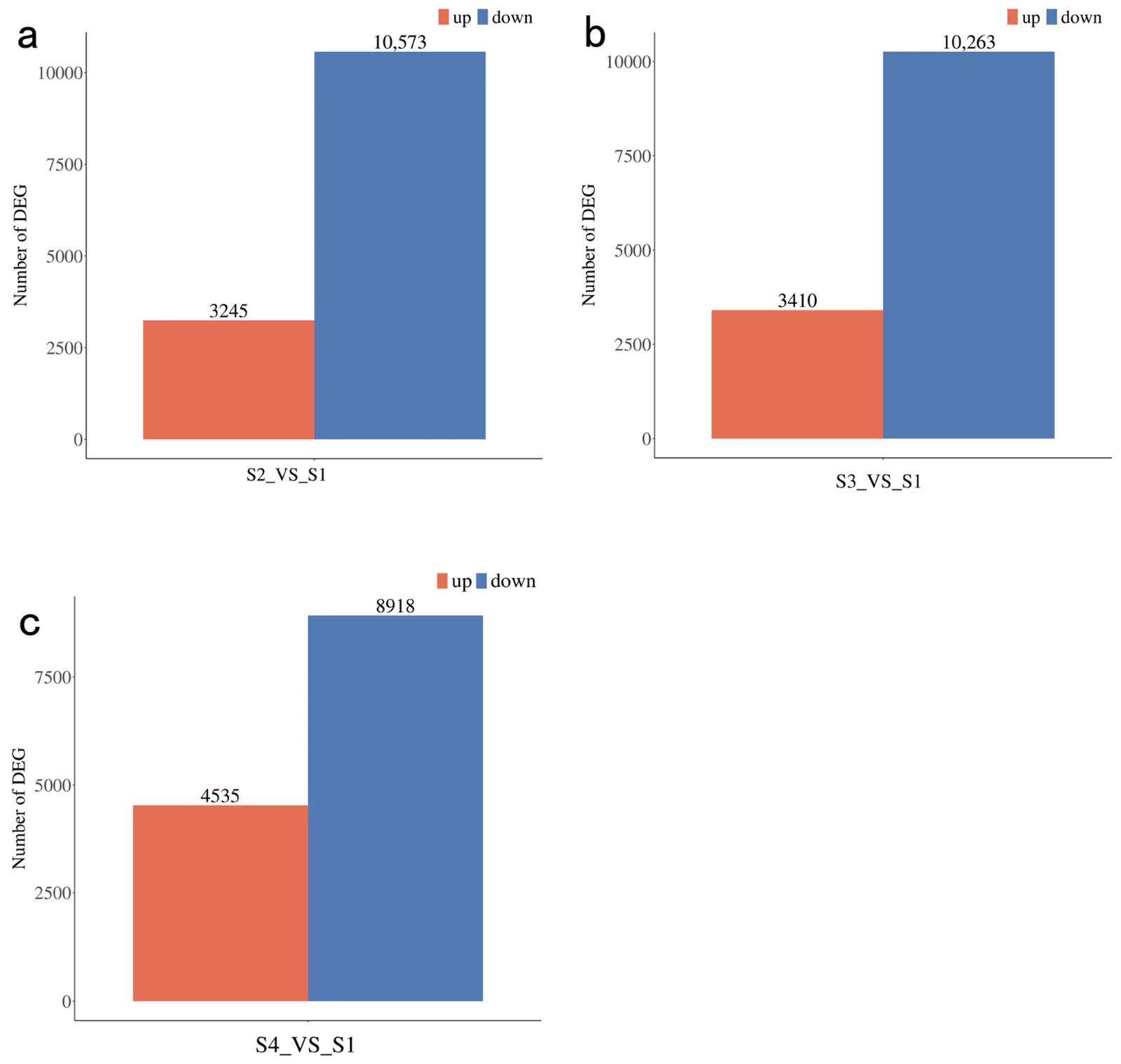
Statistics of differentially expressed genes: (**a**) S2 group; (**b**) S3 group; (**c**) S4 group.

Based on the FPKM calculation method, the statistical results for differentially expressed genes between comparison groups are shown in Figure 4.

A large number of differentially expressed genes were detected in each comparison group, indicating the widespread impact of salt stress on the transcriptome of *H. nipponensis* embryos. In the S2 and S3 salinity groups, the number of down-regulated genes (10,573 and 10,263, respectively) was significantly higher than the number of up-regulated genes (3245 and 3410, respectively), suggesting that under low- and moderate-salinity stress, embryos tend to suppress the transcriptional expression of a large number of genes.

**Figure 5:**
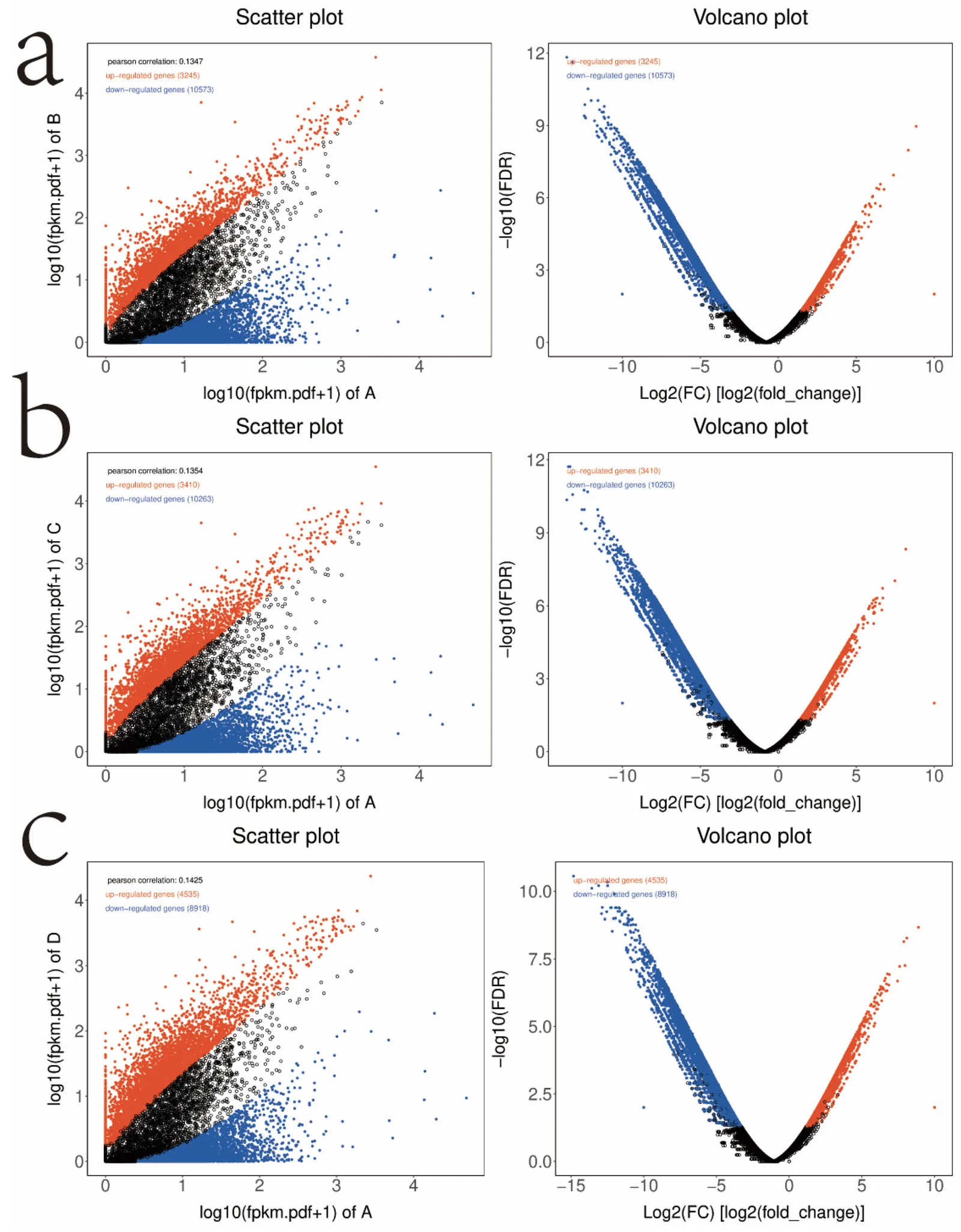
Volcano plots of differentially expressed genes in the (**a**) S2 (7‰ vs. 0‰), (**b**) S3 (14‰ vs. 0‰), and (**c**) S4 (21‰ vs. 0‰) groups. Red dots indicate significantly up-regulated genes, blue dots indicate significantly down-regulated genes, and gray dots indicate genes with no significant difference (|log_2_FC| ≥ 1 and FDR < 0.05).

Notably, in the high-salinity S4 group, the number of up-regulated genes (4535) increased significantly compared to other salinity groups. By contrast, the number of down-regulated genes (8918) relatively decreased, indicating that high-salinity stress may trigger a specific transcriptional response program distinct from that of low and moderate salinity (Figure 5).

### 3.4. Annotation of Differentially Expressed Genes

GO annotation of differentially expressed genes results in a general understanding of how these genes can be functionally classified. This analysis mainly summarized the GO annotation results of differentially expressed genes at level 2. For the S2 group, the following were included: MF (Top 10): molecular carrier activity, transporter activity, protein folding chaperone, binding, transporter binding, antioxidant activity, enzyme regulator activity, protein tag, small-molecule sensor activity, and cargo receptor activity; CC (Top 5): extracellular region, extracellular region part, intracellular, cellular anatomical entity, and organelle; and BP (Top 10): immune system process, multi-organism process, developmental process, response to stress, interspecies interaction between organisms, cellular process, multicellular organismal process, locomotion, nitrogen utilization, and metabolic process.

For the S3 group, the following were included: MF (Top 10): protein folding chaperone, transcription regulator activity, binding, antioxidant activity, small-molecule sensor activity, transporter activity, molecular function regulator, enzyme regulator activity, protein tag, and transporter binding; CC (Top 5): intracellular, cellular anatomical entity, organelle, extracellular region part, and extracellular region; and BP (Top 10): immune system process, multi-organism process, developmental process, response to stress, interspecies interaction between organisms, multicellular organismal process, locomotion, nitrogen utilization, metabolic process, and growth.

**Figure 6:**
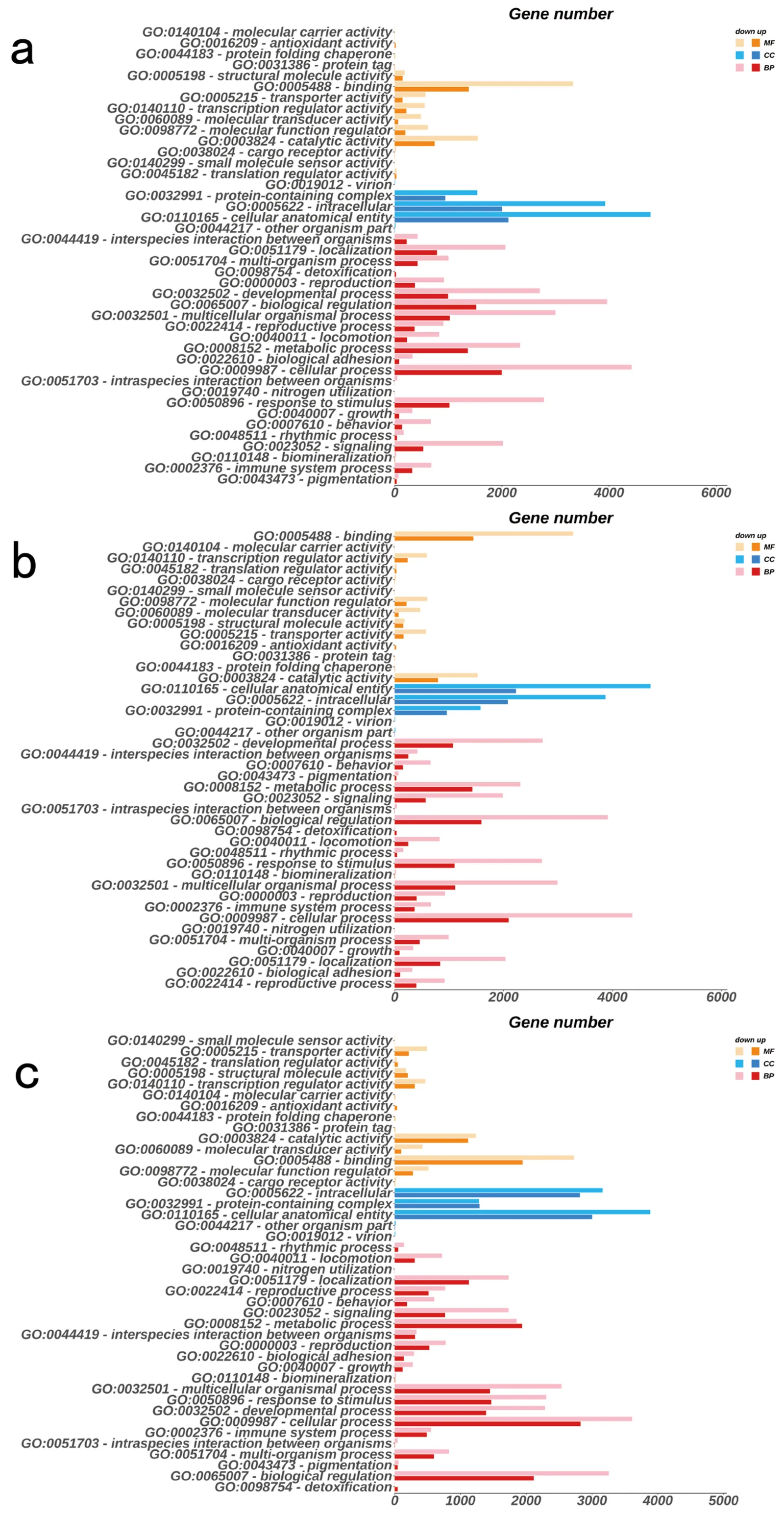
Annotation of differentially expressed genes: (**a**) S2 group; (**b**) S3 group; (**c**) S4 group.

For the S4 group, the following were included: MF (Top 10): molecular transducer activity, protein folding chaperone, transcription regulator activity, transporter activity, protein tag, binding, small-molecule sensor activity, antioxidant activity, enzyme regulator activity, and cargo receptor activity; CC (Top 5): extracellular region part, extracellular region, intracellular, cellular anatomical entity, and organelle; and BP (Top 10): immune system process, multi-organism process, developmental process, response to stress, interspecies interaction between organisms, multicellular organismal process, locomotion, nitrogen utilization, metabolic process, and growth. Details are shown in Figure 6.

### 3.5. GO Functional Enrichment Analysis

**Figure 7:**
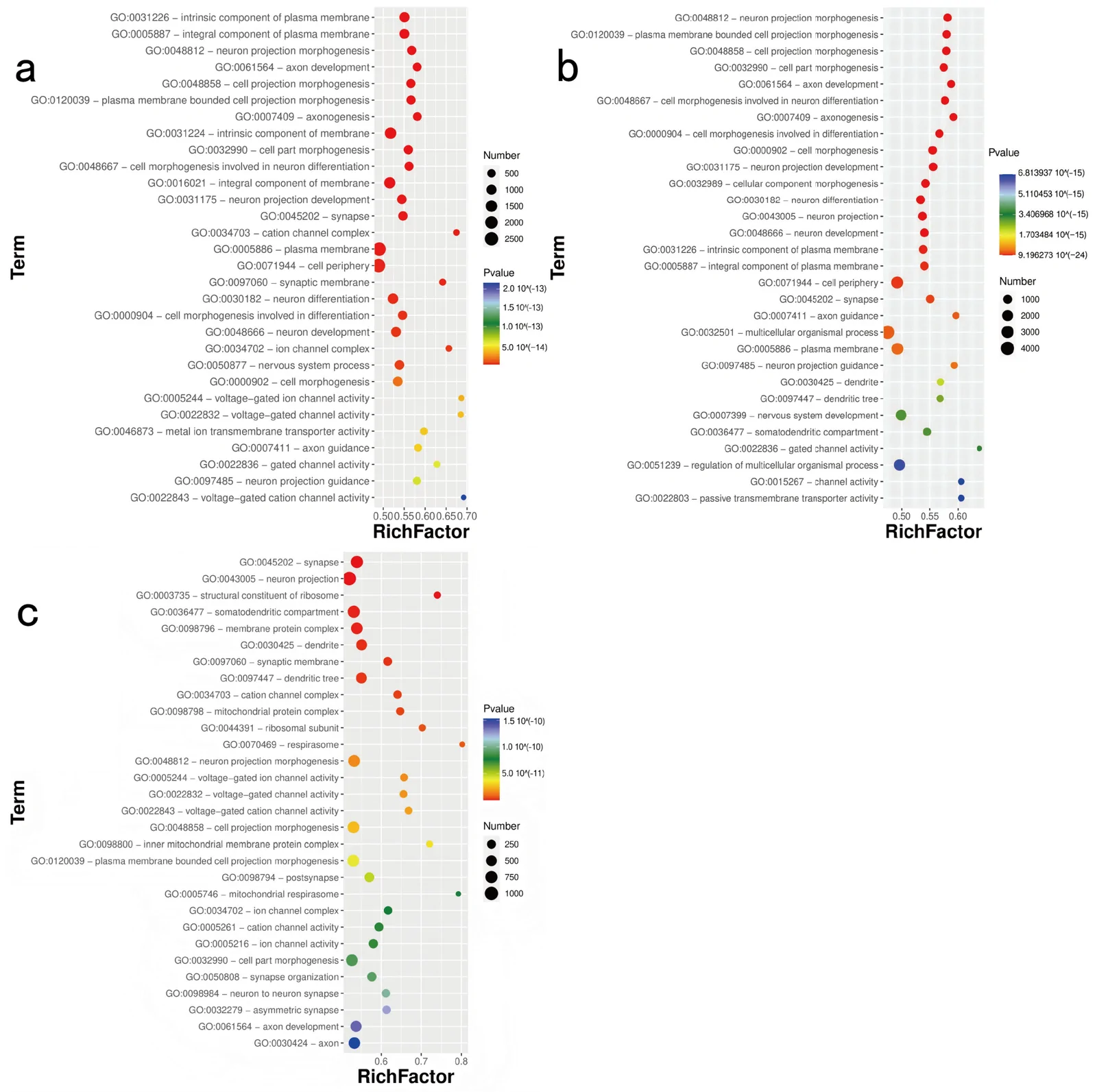
GO functional enrichment analysis: (**a**) S2 group; (**b**) S3 group; (**c**) S4 group.

GO functional enrichment analysis showed that, compared to S1, the differentially expressed genes in the S2 group were significantly enriched in terms such as dendritic tree, dendrite, inorganic cation transmembrane transporter activity, transmembrane signaling receptor activity, and metal ion transport. Compared to S1, the S3 group showed significant enrichment in GO terms mainly related to chemotaxis, inorganic molecular entity transmembrane transporter activity, cation transmembrane transporter activity, chemical synaptic transmission, and anterograde trans-synaptic signaling. In the S4 group, compared to S1, the most significantly enriched GO terms involved axonogenesis, inorganic ion transmembrane transport, dendritic tree, dendrite, and channel activity (Figure 7).

### 3.6. KEGG Pathway Enrichment Analysis

To systematically reveal the molecular regulatory mechanisms of *H. nipponensis* embryos in response to different salinity stresses, KEGG pathway enrichment analysis was performed on differentially expressed genes for each comparison group.

**Figure 8:**
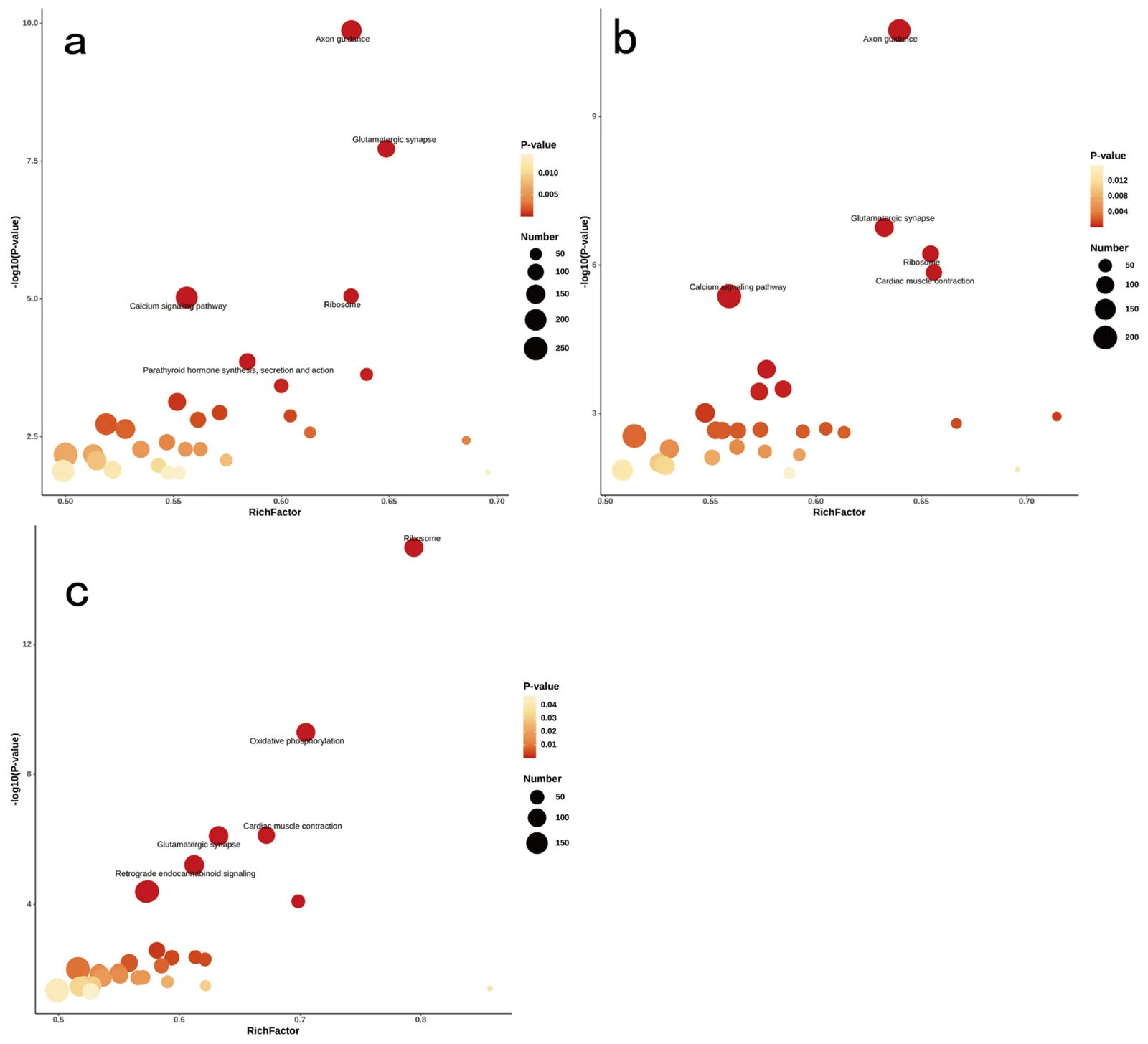
KEGG pathway enrichment analysis: (**a**) S2 group; (**b**) S3 group; (**c**) S4 group.

In embryonic tissues, compared to the S1 group, the differentially expressed genes in the S2 group were mainly enriched in axon guidance, glutamatergic synapse, calcium signaling pathway, ribosome, and parathyroid hormone synthesis, secretion, and action. The differentially expressed genes between the S3 and S1 groups were mainly enriched in axon guidance, glutamatergic synapse, ribosome, calcium signaling pathway, and cardiac muscle contraction. The differentially expressed genes between the S4 and S1 groups were mainly enriched in ribosome, oxidative phosphorylation, cardiac muscle contraction, glutamatergic synapse, and retrograde endocannabinoid signaling (Figure 8).

### 3.7. Validation of Transcriptome Data

To validate the reliability and reproducibility of the transcriptome sequencing data, we selected candidate genes for qRT-PCR validation based on the following strategy. First, we screened differentially expressed genes exhibiting significant changes (|log_2_FC| ≥ 1, FDR < 0.05) from each comparison group. From this, genes with clear functional annotations in key biological pathways, such as calcium signaling, stress response, ion transport, and redox balance, were prioritized. Finally, six representative genes were selected, including *GRPEL1* (involved in mitochondrial protein folding), *CSMD1A* (related to cell adhesion and signaling), *SPARC* (regulating extracellular matrix and calcium binding), *HNRNPA0* (involved in RNA processing and stress granule formation), *ALDOB* (a key glycolytic enzyme), and *HSPA4A* (a heat shock protein family member). These genes showed significant differential expression across multiple salinity treatment groups, suggesting important biological significance.

**Figure 9:**
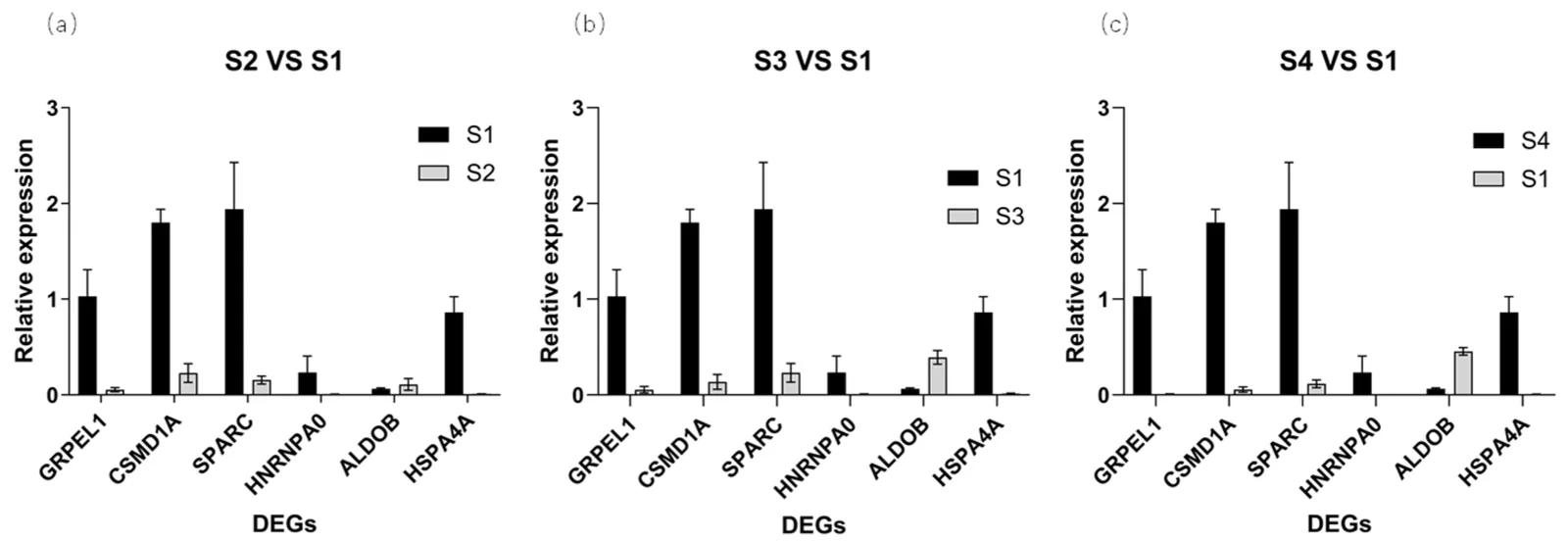
Validation of transcriptome data using qRT-PCR. (**a**) S2 (7‰) vs. S1 (0‰); (**b**) S3 (14‰) vs. S1 (0‰); (**c**) S4 (21‰) vs. S1 (0‰).

Using β-actin as the internal reference gene, qRT-PCR results showed that the expression trends of these six genes under different salinity treatments were highly consistent with the RNA-seq results (Figure 9), confirming the accuracy and reliability of the transcriptome data in this study while also verifying the expression regulation patterns of these salt stress-related functional genes.

## 4. Discussion

### 4.1. Key Findings and Key Limitations of the Experimental Design

The choice of control group (0‰) is a primary limitation of this study that must be addressed. *H. nipponensis* naturally inhabits estuarine or brackish water areas (where salinity is typically 5–7‰), and its physiological systems are likely adapted to environments with some salinity. Using freshwater (0‰) as the experimental reference point means that the control group itself may have been under hypotonic stress. Therefore, the differentially expressed genes (DEGs) observed in this study may not solely represent a response to increased salinity but could also reflect osmotic regulation as embryos transitioned from a hypotonic state to an iso-osmotic or hyperosmotic environment. This design might have confounded the transcriptomic signatures of “salinity adaptation” with “osmotic environment transition.” Specifically, the widespread transcriptional suppression observed in the 7‰ and 14‰ salinity groups may reflect the embryos’ “recovery” from the hypotonic stress of freshwater or a transition to a more suitable salinity, rather than a simple response to high-salt stress. Therefore, the conclusions regarding “salinity stress response” in this paper should be strictly interpreted as “transcriptomic reprogramming relative to the freshwater reference point,” rather than an “absolute salinity adaptation program.” Future studies should introduce a salinity baseline closer to the natural habitat (e.g., 5–7‰) as a control to distinguish true adaptation mechanisms from osmotic transition effects.

Second, the use of purified water for the control group and natural river water for the salinity treatments constitutes another significant confounding factor. Although we confirmed similar pH values (7.2 vs. 7.5), purified water contains almost none of the various mineral ions (e.g., Ca^2+^, Mg^2+^, K^+^, and Na^+^) present in specific proportions in natural river water. These differences in background ion composition could independently affect embryonic cell membrane potential, ion channel activity, and the expression of osmoregulation-related genes, regardless of salinity itself. For example, differences in extracellular Ca^2+^ concentration could directly interfere with the calcium signaling pathway’s baseline activity, one of the core pathways consistently enriched in all salinity groups in this study. Therefore, the transcriptomic differences between S2–S4 and S1 may not be caused entirely by increased salinity but may stem from systematic changes in the background water chemistry environment. This limitation necessitates caution when extrapolating the molecular conclusions of this study to natural saline–alkaline waters (especially carbonate-type or sulfate-type water bodies), as different water chemistry types may have vastly different ionic stress effects on embryos.

Third, the proportion of DEGs detected in each comparison group relative to the total number of genes was relatively high (approximately 65–67%), higher than values reported in most fish transcriptomic studies. This needs to be explained from both analytical strategy and biological background perspectives. Regarding the former, this study used a two-tier screening strategy: for the initial statistics of global DEGs, we employed a relatively lenient threshold (|log_2_FC| ≥ 1 and FDR < 0.05) to maximize the capture of all transcripts potentially involved in salinity responses, avoiding the omission of genes with smaller expression changes but significant biological significance. However, for all subsequent functional enrichment analyses (GO and KEGG), core pathway identification, and the screening and discussion of key differential genes, we consistently used a more stringent threshold (|log_2_FC| ≥ 2 and FDR < 0.05) to ensure high biological robustness of the reported pathways and genes. Biologically, the embryonic stage is a period of highly active transcriptional regulation and is extremely sensitive to environmental perturbations [21]. Simultaneously, the 0‰ freshwater control may cause hypotonic stress in this brackish-water species, compounding the differences between S2–S4 and S1 due to the dual effects of “osmotic environment transition” and “developmental plasticity,” thus amplifying the number of DEGs. Additionally, the reference genome annotation’s completeness and samples’ inherent individual heterogeneity may have contributed some statistical noise. Crucially, under the stringent threshold of |log_2_FC| ≥ 2, core pathways such as calcium signaling, axon guidance, and glutamatergic synapse remained significantly enriched, indicating that the primary biological conclusions of this study are robust. The high proportion may include some moderately changed genes, but it did not alter the qualitative results of the core pathways.

### 4.2. Non-Monotonic Response Pattern of Gene Expression

Despite the limitations of the control choice, the DEG statistics still revealed significant differences in the gene expression patterns of embryos under different salinities. A key finding was a clear non-monotonic response: in the S2 and S3 salinity stress groups, the number of down-regulated genes significantly exceeded up-regulated genes, whereas in the high-salinity S4 group, the proportion of up-regulated genes markedly increased [22,23]. We hypothesize that under moderate salinity (S2 and S3), embryos might adopt an “energy conservation” strategy by globally down-regulating the transcription of many non-essential genes, allocating limited energy and resources preferentially to physiological processes maintaining basal homeostasis and osmotic regulation. When salinity rises to 21‰, nearing their tolerance limit, this strategy cannot cope with stronger stress; the embryos initiate a series of active stress responses, leading to the specific up-regulation of many genes to counteract more severe intracellular and extracellular environmental imbalances.

### 4.3. Core Molecular Response Pathways: Calcium Signaling and Neurodevelopmental Regulation

The calcium signaling pathway was significantly enriched in all salinity treatment groups, consistent with its conserved role as a central hub for cellular stress and osmotic sensing [24,25]. Osmotic changes from salinity variation rapidly activate ion channels on the cell membrane [25], triggering fluctuations in intracellular calcium concentration, which, in turn, initiate downstream cascades regulating ion transport and aquaporin activity to maintain cell volume homeostasis. This result aligns with findings in model organisms like zebrafish embryos, indicating a universal and critical role for this pathway in fish embryos coping with osmotic stress. Additionally, pathways related to nervous system development, such as axon guidance and glutamatergic synapses, were also significantly enriched across all treatment groups. This suggests that salinity stress may affect the normal developmental program of the embryonic nervous system. As a potential stress response, embryos might regulate neurodevelopmental gene expression to adapt to or cope with physiological functional remodeling caused by osmotic changes. This finding provides new molecular clues for understanding how environmental salinity affects early neural development in fish [26,27,28,29].

### 4.4. Functional Analysis of Key Differentially Expressed Genes

To understand the molecular mechanisms by which embryos cope with salinity stress at the gene level, we focused on representative genes that exhibited significant differential expression across multiple salinity treatment groups and possessed clear functional annotations in key biological pathways. The expression changes in these genes provide direct molecular evidence for the core pathway enrichment results.

#### 4.4.1. Calcium Signaling Pathway-Related Genes

As a core pathway consistently enriched across all treatment groups, calcium signaling pathway activation is supported by changes in the expression of several key differential genes. For instance, the *SPARC* (secreted protein acidic and cysteine-rich) gene was significantly up-regulated in multiple salinity groups. *SPARC* is a calcium-binding glycoprotein involved in extracellular matrix remodeling, cell adhesion, and calcium homeostasis regulation. Its up-regulation may reflect an adaptive response of embryos to maintain tissue homeostasis by modulating the extracellular matrix and calcium signaling in response to osmotic changes. This gene’s changing expression provides good gene–pathway corroboration with the calcium enrichment observed at the pathway level.

#### 4.4.2. Stress Response and Protein Homeostasis-Related Genes

As an environmental stressor, salinity stress inevitably triggers an imbalance in cellular protein homeostasis and stress responses. *HSPA4A* was significantly up-regulated in the 21‰ high-salinity group; it is a classic molecular marker of activated cellular stress response [30]. *HSPA4A*, as a molecular chaperone, participates in the recognition, repair, and degradation of misfolded proteins; its up-regulation indicates that high salt stress led to an accumulation of protein damage in embryonic cells. Changes in *GRPEL1* expression, which encodes a cofactor for mitochondrial protein folding, suggest that mitochondrial protein homeostasis is also impacted by high salt stress. Coordinated changes in these stress-responsive genes align with the inference that cellular stress programs are fully activated under high salinity.

#### 4.4.3. Energy Metabolism-Related Genes

In the S2 and S3 salinity stress groups, we observed global transcriptional suppression, suggesting that embryos have a possible energy conservation strategy. *ALDOB* (fructose-bisphosphate aldolase B), a key enzyme in the glycolytic pathway, is primarily expressed in the liver and kidneys of fish and participates in glucose metabolism. In this study, *ALDOB* showed significant down-regulation across different salinity groups. This change may relate to an overall down-regulation of the embryonic metabolic rate under salinity stress [31], providing gene-level evidence for the “energy conservation” hypothesis. The widespread suppression of metabolism-related genes aligns with the transcriptomic feature of massive gene down-regulation observed under moderate salinity.

#### 4.4.4. Neurodevelopment-Related Genes

KEGG enrichment analysis revealed that neurodevelopmental pathways, such as axon guidance and glutamatergic synapse, were significantly enriched in all treatment groups. This is closely linked to expression changes in genes like *CSMD1A*. *CSMD1A* (CUB and Sushi multiple domains 1) is a transmembrane protein highly expressed in the nervous system, involved in neuronal development, synapse formation, and complement system regulation. Its differential expression across salinities suggests that salinity stress may interfere with normal neurodevelopmental programs by affecting their expression. Concurrently, *HNRNPA0* (heterogeneous nuclear ribonucleoprotein A0), which is an RNA-binding protein involved in pre-mRNA processing and stress granule formation, may reflect post-transcriptional stress regulation in neuronal cells under salinity stress [32].

In summary, the changing patterns of these key differentially expressed genes demonstrate high-level gene–pathway consistency with core findings at the pathway level (calcium signaling activation, enhanced stress response, metabolic inhibition, and neurodevelopmental regulation), providing finer-grained evidence for the molecular regulatory network of *H. nipponensis* embryos responding to salinity stress.

## 5. Conclusions

This study definitively establishes that the upper salinity tolerance limit for pond smelt embryos is between 21‰ and 28‰: the hatching rate decreased to (40.3±6.1)% at 21‰, and hatching failed completely at 28‰ and above. Transcriptome analysis revealed that embryos adopt a global transcriptional suppression strategy at moderate salinities (7‰, 14‰) (with the number of down-regulated genes being approximately three times that of up-regulated genes), while activating an active stress program at high salinity (21‰) (up-regulated genes increased to 4535). The calcium signaling pathway was consistently enriched in all salinity groups. The significant changes in axon guidance and glutamatergic synapse pathways suggest neurodevelopment may be a sensitive target of salinity effects. Expression changes in key genes such as *hspa4a*, *sparc*, and *aldob* molecularly corroborate the coordinated responses of stress, calcium homeostasis, and metabolic reprogramming. Importantly, we emphasize that these conclusions are based on a comparative framework using freshwater (0‰) as the experimental reference point, which may not be the optimal physiological baseline for this brackish-water species. Therefore, the transcriptomic changes revealed in this study should be understood as response characteristics during the transfer of embryos from a hypotonic to a hypertonic environment, rather than a pure “salinity adaptation” program.

In summary, pond smelt embryos have the molecular basis for normal development in waters with salinity up to 14‰, but higher salinity will severely restrict their hatching success rate. This study provides key molecular evidence for evaluating this species’ aquaculture and enhancement potential in saline–alkaline waters [33].

## Figures and Tables

**Table 1:** Primer sequence information for real-time fluorescence quantitative PCR amplification.

Gene	Forward Primer	Reverse Primer
*GRPEL1*	5′ ACAGGGTTCCATGTCC 3′	5′ TTTGAAGCCGTGCGCGCTGACGGCA 3′
*CSMD1A*	5′ CTCTGTTGCAGTGGTGGGT 3′	5′ TCCCCGCACTCCCGCTCCTCAGTCA 3′
*SPARC*	5′ GAATCCCTGTCTGAACCACCACT 3′	5′ ATCAACCTCACACACCTTACCCTT 3′
*HNRNPA0*	5′ TTTTTGTCGGTGGATTGAACGTC 3′	5′ GCATCTCTTGCTTGGTGAGAGCTT 3′
*ALDOB*	5′ CTCACCAGTTCCCATCCCTCTCTCC 3′	5′ CGTCGGTTCTCCTCTGTGTTCTCCA 3′
*HSPA4A*	5′ GGACTGAGTGATTGTGAGGTGTTCC 3′	5′ TGGTCTGTCTTCTTCTCCTGTTTGG 3′
*β-actin*	5′ TGAAATCGCCGCACTGGTT 3′	5′ TCCGTGCTCGATGGGGTACT 3′

## Data Availability

The raw RNA-seq data generated in this study have been deposited in the NCBI Sequence Read Archive (SRA) under BioProject accession number PRJNA1495305. The data are publicly available at: https://dataview.ncbi.nlm.nih.gov/object/PRJNA1495305?reviewer=fq1hg943iudfmp4llkhvlm30jj (accessed on 13 September 2026). All other data are contained within the manuscript.

## Abbreviations

The following abbreviations are used in this manuscript:

DEGs	Differentially Expressed Genes
GO	Gene Ontology
KEGG	Kyoto Encyclopedia of Genes and Genomes
FPKM	Fragments Per Kilobase of Transcript Per Million Mapped Reads
TPM	Transcripts Per Million
FDR	False Discovery Rate
PCA	Principal Component Analysis
RNA-seq	RNA Sequencing
qRT-PCR	Quantitative Real-Time Reverse Transcription PCR
cDNA	Complementary DNA
DNA	Deoxyribonucleic Acid
MAPK	Mitogen-Activated Protein Kinase
CC	Cellular Component
BP	Biological Process
NCBI	National Center for Biotechnology Information
